# *Trans*-Activator Binding Site Context in RCNMV Modulates Subgenomic mRNA Transcription

**DOI:** 10.3390/v13112252

**Published:** 2021-11-10

**Authors:** Jennifer S. H. Im, Laura R. Newburn, Gregory Kent, K. Andrew White

**Affiliations:** Department of Biology, York University, Toronto, ON M3J 1P3, Canada; imjenim@my.yorku.ca (J.S.H.I.); lnewburn@yorku.ca (L.R.N.); greg.kent@mail.utoronto.ca (G.K.)

**Keywords:** RNA virus, plant virus, *Tombusviridae*, tombusvirus, dianthovirus, RNA–RNA, RNA structure, RNA folding, subgenomic mRNA, sgRNA, RdRp, transcription

## Abstract

Many positive-sense RNA viruses transcribe subgenomic (sg) mRNAs during infections that template the translation of a subset of viral proteins. Red clover necrotic mosaic virus (RCNMV) expresses its capsid protein through the transcription of a sg mRNA from RNA1 genome segment. This transcription event is activated by an RNA structure formed by base pairing between a *trans*-activator (TA) in RNA2 and a *trans*-activator binding site (TABS) in RNA1. In this study, the impact of the structural context of the TABS in RNA1 on the TA–TABS interaction and sg mRNA transcription was investigated using in vitro and in vivo approaches. The results (i) generated RNA secondary structure models for the TA and TABS, (ii) revealed that the TABS is partially base paired with proximal upstream sequences, which limits TA access, (iii) demonstrated that the aforementioned intra-RNA1 base pairing involving the TABS modulates the TA–TABS interaction in vitro and sg mRNA levels during infections, and (iv) revealed that the TABS in RNA1 can be modified to mediate sg mRNA transcription in a TA-independent manner. These findings advance our understanding of transcriptional regulation in RCNMV and provide novel insights into the origin of the TA–TABS interaction.

## 1. Introduction

Members of the positive-sense RNA virus family *Tombusviridae* [1] express certain encoded proteins via the production of subgenomic (sg) mRNAs [2,3,4,5,6,7,8]. These smaller viral messages are 3′-coterminal with their cognate viral genomes and encode open reading frames (ORFs) that are translationally silent within the full-length genomic context [9]. Tombusvirids utilize a premature termination mechanism for sg mRNA transcription, where the viral RNA-dependent RNA polymerase (RdRp) terminates internally at an attenuation RNA structure during negative-strand RNA synthesis of the viral genome [10]. The resulting truncated minus strand contains a promoter at its 3′ end that is utilized by the RdRp to transcribe a corresponding sg mRNA. Attenuation RNA structures are formed by localized genomic sequences [3,6], long-distance RNA–RNA interactions [11,12], or RNA–RNA interactions between genome segments (i.e., *trans*-interactions) [13], and may adopt simple RNA secondary structures [3,6] or elaborate higher-order RNA conformations created by genome-level folding [12]. Common to all of these structures is a base-paired region (typically 6 to 10 bps in length) positioned approximately 2 to 5 nts upstream from the RdRp stall site [6].

**Figure 1:**
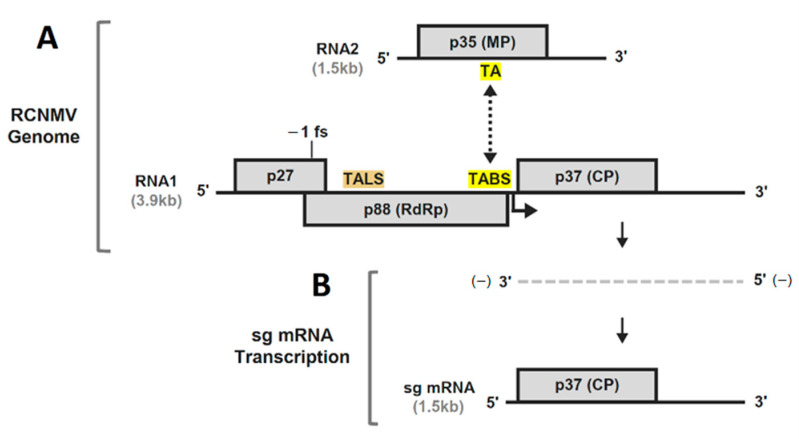
RCNMV genome organization and subgenomic mRNA synthesis. (**A**) Structures of RCNMV RNA1 and RNA2 genomes. Nucleotide lengths are shown in kilobases (kb). Encoded proteins are shown as grey boxes with their corresponding sizes indicated in kilodaltons. The positions of the TA in RNA2 and the TABS in RNA1 are highlighted in yellow, and the TALS in RNA1 is highlighted in tan. The black dashed arrow signifies the interaction between complementary TA and TABS sequences. The location of the transcription initiation site of the sg mRNA in RNA1 is indicated by the angled arrow. (**B**) Subgenomic mRNA transcription from RNA1. The *trans*-TA–TABS interaction functions as an attenuating RNA signal for premature termination during minus (−)-strand synthesis of RNA1, resulting in a truncated (−)-strand, shown as a grey dashed line, which then serves as the template for transcription of the subgenomic mRNA.

Red clover necrotic mosaic virus (genus *Dianthovirus*, family *Tombusviridae*) possesses a segmented plus-strand RNA genome composed of RNA1 (3.9 kb) and RNA2 (1.5 kb) (Figure 1A) [14]. RNA1 encodes accessory replication protein p27, and translational frameshifting near the end of its ORF produces p88, the RdRp [15]. The coat protein (CP) is the third ORF encoded in RNA1 and is expressed via transcription of a sg mRNA (1.5 kb) during infections (Figure 1B) [13]. RNA2 encodes a single protein, p35, the cell-to-cell movement protein (Figure 1A) [16].

**Figure 2:**
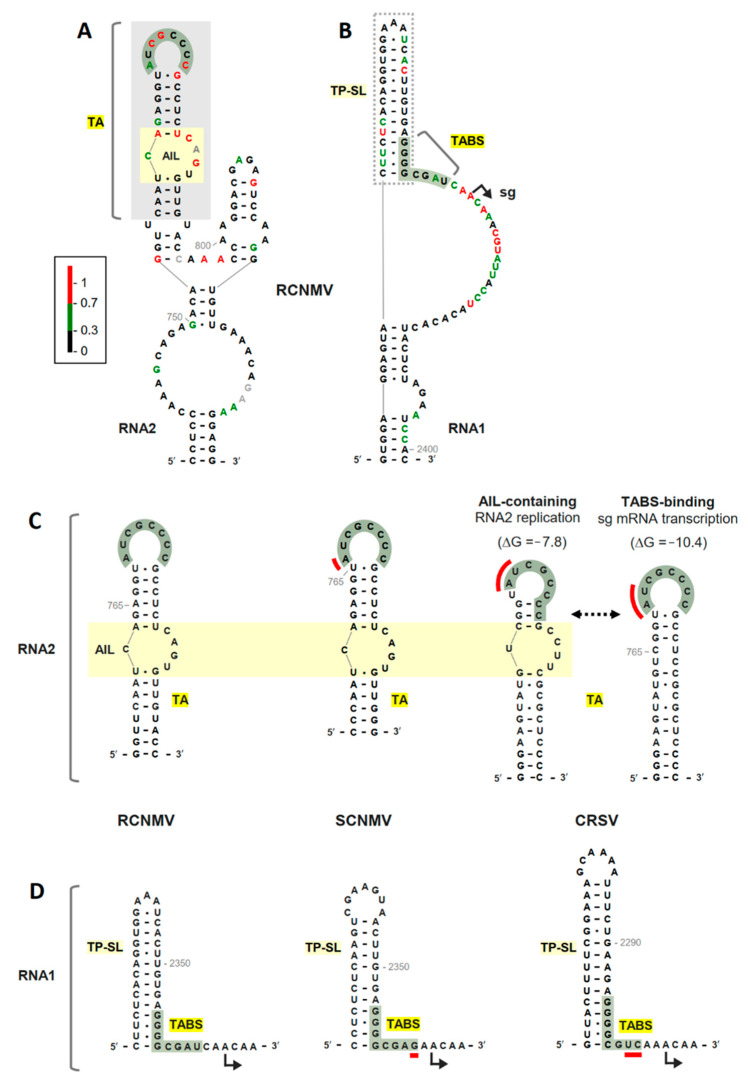
RNA secondary structure models of the TA and TABS in dianthoviruses. (**A**) SHAPE-guided model of TA-containing region of RCNMV RNA2. The TA and AIL structures are denoted by grey and yellow shaded boxes, respectively. The green shading depicts the terminal loop of the TA that is complementary to the TABS. Relative reactivity of nucleotides are depicted as red, green, or black bases, indicating high, moderate, and low reactivity, respectively. Grey bases denote null reactivity values. (**B**) SHAPE-guided model of TABS-containing region of RCNMV RNA1. Relative nucleotide reactivities are as described in panel A. The TABS sequence is depicted by green shading and the TP-SL structure is shown in the dotted grey box. The position of the transcription initiation site for the subgenomic mRNA (sg) is shown as an angled arrow. (**C**) Comparative secondary structure analysis of RNA2 TA in RCNMV, SCNMV, and CRSV. The dotted arrow depicts two conformations of the CRSV TA structures with their corresponding computationally predicted free energies (kcal/mol). The red lines indicate mismatches in the TA–TABS interaction. (**D**) Comparative secondary structure analysis of the RNA1 TABS and TP-SL structures in RCNMV, SCNMV, and CRSV.

Transcription of the RCNMV sg mRNA involves a *trans*-acting base pairing interaction between short sequences located near the end of the RdRp-coding region in RNA1 (termed the *trans*-activator binding site or TABS) and at a central position within the movement protein ORF in RNA2 (termed the *trans*-activator or TA) (Figure 1A) [13]. The TA in RNA2 is an RNA stem–loop (SL) structure containing an 8 nt terminal loop that base pairs with the complementary 8 nt long TABS sequence in RNA1 that is located two nucleotides upstream from the transcription initiation site (Figure 2A,B). The RNA structure formed by this interaction blocks the RdRp’s progress during minus-strand synthesis of RNA1, generating the minus-strand RNA template for sg mRNA transcription (Figure 1B). The TA has also been implicated as a packaging signal [17,18] and its interaction with TABS facilitates co-packaging of the two viral segments [19]. Additionally, the TA is required for the replication of RNA2 [20], a function that is mediated primarily by an asymmetric internal loop (AIL) (Figure 2A), which is also present in a TA-like structure (TALS) located in RNA1 that is essential for its replication (Figure 1A) [21]. The multifunctional TA has been the focus of several studies [13,17,18,19,20]; however, the TABS has received much less attention. Here, we examined the structural context of the TABS in relation to its role in sg mRNA transcription.

## 2. Materials and Methods

### 2.1. Viral Constructs

The full-length cDNA clones of wild-type RCNMV RNA1 and RNA2 genome segments (kindly provided by Tim Sit and Steven Lommel) [13] were used to generate all RCNMV constructs used in this study. RCNMV mutants were derived using standard site-directed PCR-based mutagenesis, using the Q5 High-Fidelity DNA Polymerase kit (NEB). All mutant viral genome constructs were sequenced across the PCR inserts containing modifications to ensure that only the intended change was present. Specific changes introduced into the wild-type RCNMV genomes are shown in the figures.

### 2.2. Preparation of Infectious Viral RNAs

Uncapped viral RNAs were in vitro-transcribed from SmaI-linearized RCNMV RNA1 and RNA2 constructs using the T7-FlashScribeTM transcription kit (Cellscript) as described previously [22]. RNA concentrations were measured by spectrophotometry (DU800 UV/Visible Spectrophotometer, Beckman Coulter), and transcript integrity was verified by agarose gel electrophoresis.

### 2.3. SHAPE RNA Secondary Structure Analysis

SHAPE (selective 2′-hydroxyl acylation analyzed by primer extension) was performed on the TABS and TA regions of full-length RNA1 and RNA2 genomes, respectively, as described previously [23]. Briefly, full-length in vitro-transcribed genomic RNAs were refolded and subsequently treated with 1-methyl-7-nitroisatoic anhydride. The reaction products were reverse transcribed using Superscript IV reverse transcriptase (Invitrogen, Waltham, MA, USA). Fluorescently labeled primers complementary to RNA1 (nt 2501–2527) and RNA2 (nt 917–948) were used to map the secondary structures of the regions of interest. Raw fluorescence intensity data were analyzed using ShapeFinder [24] and nucleotide reactivity data were normalized against the average of the top ten peak intensities. SHAPE reactions were performed twice and averaged nucleotide reactivities were used. The RNA structure web server was used to integrate reactivities with thermodynamic prediction to generate secondary structure models [25]. The averages of normalized SHAPE reactivities from two independent experiments were used as folding constraints in RNAstructure (slope value = 1.8 kcal/mol and intercept value = −0.6 kcal/mol) to deduce an RNA secondary structure for the TA and TABS. The RNA structures presented for RCNMV were predicted both through folding of local regions of interest containing the TA and TABS and global folding of entire genomes; the structures generated were consistent in both contexts. RNA secondary structure figures were generated using RNA2Drawer software (version 6.3) [26].

### 2.4. Protoplast Transfections and Viral RNA Analysis

Cucumber protoplasts were isolated from 6-day-old cotyledon leaves and isolated as described previously [22]. Approximately 3 × 10^5^ protoplasts were transfected with infectious viral transcripts (3 μg of RNA1 and/or 1 μg of RNA2) using PEG-CaCl_2_. Transfected protoplasts were incubated under constant light (60 watt) at 17 °C for 22 h. Total nucleic acids were extracted using phenol-chloroform isoamyl alcohol purification. Following ethanol precipitation, one-sixth of the total volume was separated in 2% agarose gels and products were transferred to nylon membrane. Plus-strand viral RNA accumulation was detected via northern blotting using ^32^P-ATP radiolabeled DNA oligonucleotide probes that are complementary to the 3′ end of RNA1 (nt 3704–3724, and 3789–3809) and RNA2 (nt 1222–1242, and 1321–1342), as described previously [21]. RNA1 and RNA2 were probed sequentially, as RNA2 and the sg mRNA co-migrate. For minus strand detection, total nucleic acids from protoplast transfections were separated in 1.4% agarose gels after glyoxal denaturation as described previously [27]. Minus-sense viral RNA accumulation was detected via northern blotting using ^32^P-UTP labeled riboprobes (nt 3621–3890) complementary to the 5′ end of negative-sensed RNA1. Viral RNA accumulation was measured by radioanalytical scanning using a Typhoon FLA 9500 variable mode imager (GE Healthcare, Chicago, IL, USA) and quantified by Quantity One Software (Bio-Rad). Infections and northern blotting analyses were repeated for a minimum of three trials to generate averages and corresponding standard error of the means (SEMs).

### 2.5. Electrophoretic Mobility Shift Assay (EMSA)

Full-length RNA1 and RNA2 genomes subjected to EMSA were in vitro-transcribed from the viral constructs described above. EMSAs were performed using modified methods from a previously described protocol [28]. Briefly, individual, or mixtures of RNA1 and RNA2 genomes (10 pmol each) were heated at 94 °C for 3 min and combined with RNA binding buffer (5 mM HEPES, 6 mM MgCl_2_, 100 mM KCl, 3.8% glycerol) [23,28,29]. Reactions were incubated at 37 °C for 30 min and snap-cooled on ice for 2 min. Samples were combined with sterile glycerol (10% final concentration), and the entire contents were separated by agarose gel electrophoresis in TBM buffer (45 mM Tris, pH 8.3, 43 mM boric acid and 2 mM MgCl_2_) [29]. Gels were stained with ethidium bromide (1 mg/mL) [23] and imaged using a Typhoon FLA 9500 scanner (GE Healthcare). RNA bands were quantified using the Quantity One software. Relative RNA1–RNA2 binding efficiencies are presented as a percentage of shifted RNA1 by quantifying the unbound levels of RNA1 in the RNA1-only lanes and comparing it against the corresponding remaining unbound levels in mixtures with RNA2. Each EMSA experiment was conducted three times independently, to generate averages and corresponding SEMs.

## 3. Results and Discussion

### 3.1. RNA Secondary Structure Analyses of the TA and TABS

Previously, nuclear magnetic resonance spectroscopy and computer modelling was used to generate a structural model for the 34 nt long TA bound to an 8 nt TABS oligoribonucleotide [30]. The same study also performed binding affinity analysis with these oligoribonucleotides. In both cases, the structure of the TABS within its RNA1 genomic context was not considered. To our knowledge, there have not been any structural studies that have examined the TABS, or the TA, in the natural contexts of their respective viral genome segments. To address this issue, we performed solution structure mapping by SHAPE in the regions encompassing the TA in full-length RNA2 and the TABS in full-length RNA1. The reactivity data generated were then used to generate SHAPE-guided thermodynamics-based models for both RNA elements as described in Material and Methods [25].

The results predicted the presence of the TA as part of a multihelical RNA structure (Figure 2A). Within the TA, highly reactive (i.e., flexible and likely single-stranded) residues mapped within and adjacent to the terminal loop and asymmetrical internal loop (AIL). The structure also included a smaller 3′-adjacent SL and a lower stem interrupted by a large internal loop. The low reactivity for the two halves of the internal loop suggests that they may either interact noncanonically with each other or have partner sequences outside of this region. Comparison of the RCNMV TA with the TAs in two other sequenced dianthoviruses, Sweet clover necrotic mosaic virus (SCNMV) and Carnation ringspot virus (CRSV), revealed corresponding terminal loops and AILs (Figure 2C).

Interestingly, examination of the TA for CRSV revealed that, in order for it to include an AIL and a terminal loop complementary to its TABS, it would have to adopt two mutually-exclusive conformations (Figure 2C). Notably, these alternate TA structures could provide a potential mechanism to coordinate sg mRNA transcription and replication of RNA2, because when the TA is bound to the TABS and mediating sg mRNA transcription, the simultaneous synthesis of a minus strand for RNA2 by the RdRp would disrupt the TA–TABS interaction and halt transcription [21]. Accordingly, inhibition of RNA2 replication during sg mRNA transcription would be beneficial, and this could be achieved by transcription being activated with the TABS-binding TA conformation (Figure 2C), which cannot concurrently harbor the AIL that is critical for RNA2 replication [21]. Conversely, since RCNMV and SCNMV have single TA conformations (containing both the AIL and terminal loop complementary to the TA), they would need to utilize a different strategy to coordinate these opposing processes.

Examination of the predicted structural context of the TABS in RCNMV RNA1 indicated that its 5′-half is base paired within the lower portion of a 5′-adjacent SL, termed TABS-proximal SL (TP-SL) (Figure 2B). The remainder of the TABS sequence also exhibited low reactivity, but the modeling depicted it as single stranded along with the more reactive 3′-adjacent sequence. More distal flanking sequences formed a lower stem interrupted by a bulge. When the local structural context of the RCNMV TABS was compared to those in SCNMV and CRSV, corresponding TP-SLs were observed that were formed with different sequences (Figure 2D). Additionally, as in RCNMV, the other TP-SLs included pairing of their lower regions with the 5′-halves of their respective TABSs. Curiously, both SCNMV and CRSV had substitutions at the 3′ end of their TABS, which would cause mismatches with the corresponding partner sequences in their TAs (Figure 2C,D, red lines). Consequently, the TA–TABS interactions in SCNMV and CRSV likely involve incomplete or noncanonical base pairing, which could act to fine-tune the interaction and its related activities.

### 3.2. The TABS Structural Context Modulates the TA–TABS Interaction and sg mRNA Transcription

**Figure 3:**
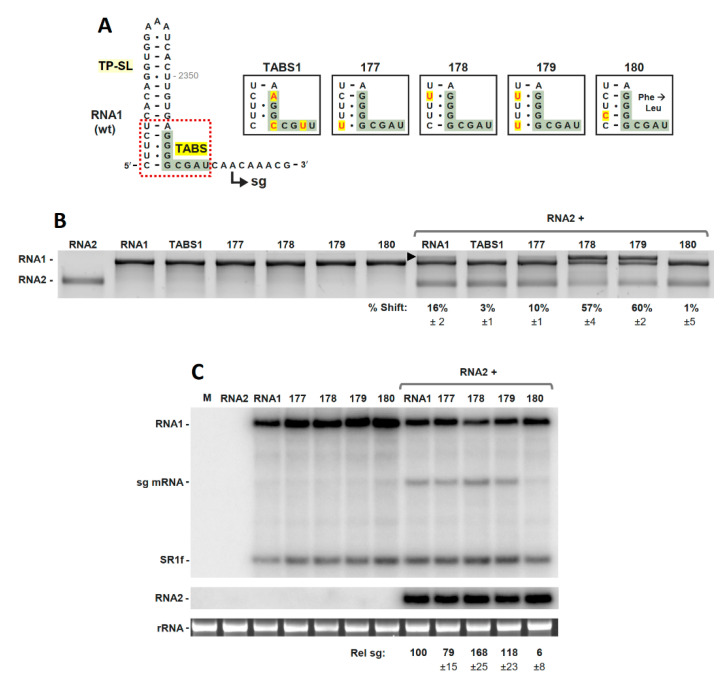
Mutational analysis of the TP-SL/TABS pairing in RCNMV RNA1. (**A**) Wild-type RCNMV RNA1 and mutants with nucleotide substitutions depicted in red with yellow highlighting. (**B**) RNA–RNA electrophoretic mobility shift assay of wt RNA1 and RNA1 mutants with wt RNA2 performed in 3% agarose gels stained with ethidium bromide. The position of RNA1–RNA2 complexes is indicted by an arrowhead. The contents of each lane are indicated above the gel and the percentages for shifted RNA1 are shown with SEM (n = 3). (**C**) Northern blot analysis of plus-strand genome accumulation from wt RNA1 and mutants transfected alone or with RNA2. RNA was isolated from protoplasts 22 h post transfection. The contents of each lane are indicated above the blot. The positions of the RNA1 and RNA2 genome segments, the subgenomic mRNA, and a stable degradation product of RNA1 (SR1f) are indicated on the left. Loading controls (rRNA) are also shown. In each coinfection, the level of sg mRNA accumulation was calculated relative to the accumulation level of its cognate RNA1. The relative levels of sg mRNA (Rel sg) and RNA2 (Rel RNA2) are shown below with SEM (n = 3).

The observation that the 5′-half of the TABS is predicted to be paired within the base of the TP-SL in all three dianthoviruses suggested that the TABS, within its genomic context, may not be readily available for pairing with the TA (Figure 2D). This inaccessibility could explain why an 8 nt long oligoribinucleotide of the TABS bound efficiently to the TA in vitro [30], while similar attempts with the full-length RCNMV genome segments were unsuccessful [19]. To test the possibility of a TA–TABS-mediated intergenomic interaction in vitro, RNA–RNA EMSA analysis was performed with full-length RNA1 and RNA2 at a one-to-one molar ratio. A modest, but consistently detectable level of complex formation (16%) was observed for wt RNA1 and wt RNA2, which was reduced to background levels (3%) when three mismatches were introduced into the TABS in RNA1 mutant TABS1 (Figure 3A,B). Reducing the predicted stability of the TP-SL/TABS pairing in RNA1 mutants 177, 178 and 179, by converting CG base pairs in TP-SL to UG pairs, resulted in decreased binding for 177 (10%), and increased binding for 178 (57%) and 179 (60%) (Figure 3A,B). These results indicate that the CG to UG change at the end of TP-SL did not assist the interaction, while a comparable change further in was beneficial (Figure 3B). In contrast, strengthening the TP-SL/TABS pairing in RNA1 mutant 180 effectively abolished binding (1%) (Figure 3A,B). These in vitro results were generated using equivalent amounts of RNA1 and RNA2; however, the absolute and relative levels of these genome segments during infections, which would influence the efficiency and timing of complex formation, is unknown. Collectively, our EMSA findings (i) provide physical evidence that complements the genetic evidence [13] for a TA–TABS interaction between RNA1 and RNA2, (ii) support a structural model that places the 5′-half of the TABS within TP-SL, and (iii) demonstrate that the stability of the TP-SL/TABS pairing correlates inversely with the formation of the TA–TABS-mediated RNA1–RNA2 interaction.

To determine if the RNA1 mutations that altered TP-SL/TABS pairing also affected sg mRNA levels during infections, these mutants were transfected into protoplasts in the absence or presence of RNA2. In each coinfection, the level of sg mRNA accumulation was calculated relative to the accumulation level of its cognate RNA1. Consistent with the EMSA results, co-transfections of RNA2 with RNA1 mutant 177 moderately reduced relative sg mRNA levels (79%), with mutant 178 notably enhanced levels (168%), and with mutant 180 essentially abolished accumulation (3%) (Figure 3C). Co-transfections with RNA1 mutant 179, containing both of the modifications present in mutants 177 and 178, exhibited sg mRNA levels intermediate of the latter two (118%) (Figure 3C). Thus, in protoplast infections, the relative sg mRNA levels observed for these mutants were consistent. However, in in vitro binding assays, mutant 179 exhibited similar binding activity to mutant 178 (Figure 3B). This discrepancy, equivalent binding activities yet different relative levels of sg mRNA accumulation, indicates that the complexity of the in vivo environment is not always be reflected by the in vitro assay. Regardless, the collective results from these two related analyses support the logical concept that TABS accessibility, as determined by its genomic context, is able to modulate both the TA–TABS interaction and sg mRNA transcription. Accordingly, RCNMV may have used this feature to adjust the timing and amount of sg mRNA for optimized infections. Indeed, the different levels of TP-SL/TABS pairing seen in SCNMV and CRSV are consistent with this concept (Figure 2D).

### 3.3. The TABS Can Be Converted into a TA-Independent, Cis-Acting, Attenuation RNA Structure

**Figure 4:**
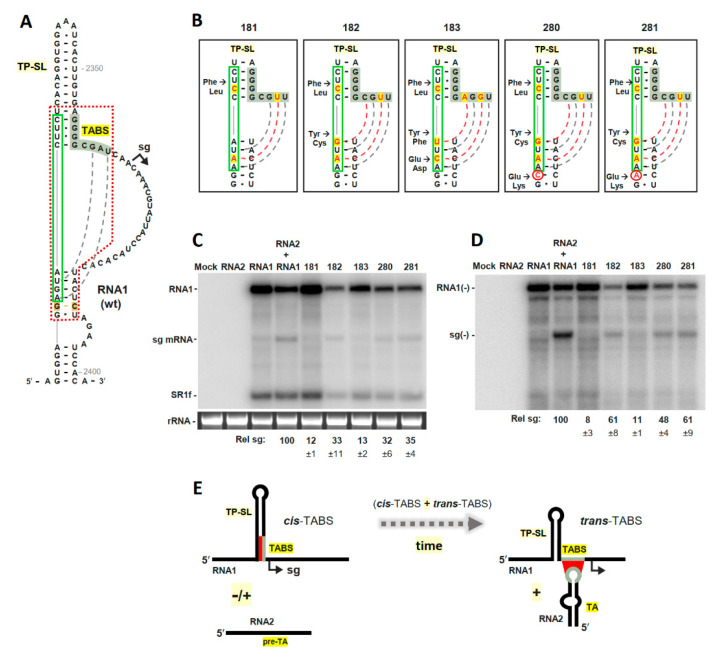
Modifying the structural context of the TABS in RCNMV RNA1. (**A**) Structure of the TP-SL/TABS region in wt RCNMV RNA1. The potential partner sequence for the TABS is boxed in green, with existing base pairs indicated by the grey dotted line. The red dashed line defines the regions shown in (**B**). (**B**) RNA1 TP-SL/TABS mutants with nucleotide substitutions are depicted in red with yellow highlighting. Additional substitution mutations made in 182 (to generate 280 and 281) are circled in red. Intended base-pairing is shown by dotted lines for new (red) and existing (grey) partners. Amino acid changes are indicated to the left. (**C**) Northern blot analysis of wt plus-strand RNA1 and TP-SL/TABS mutants in plant protoplast transfections (n = 3). Loading controls (rRNA) are also shown. (**D**) Northern blot analysis of minus-strand RNA1 accumulation in plant protoplast transfections (n = 3). (**E**) Simplified depiction of the evolution of sg mRNA transcription from RNA1. Base pairing leading to sg mRNA transcription is depicted in red. See text for details.

The presence of the 5′-half of the TABS within TP-SL prompted us to consider whether further pairing of the 3′-half of the TABS with the sequence immediately 5′-proximal to TP-SL could potentially generate a SL-based, *cis*-acting, attenuation RNA structure. Indeed, we proposed previously that RNA1 likely expressed its sg mRNA via a simpler *cis*-acting attenuation RNA structure before evolving to its current more sophisticated *trans*-mechanism [21]. Examination of the TP-SL/TABS region revealed that the sixth and eighth nucleotides in the 3′-half of the TABS could base pair, via UG and AU, respectively, with corresponding nucleotides located just in front of TP-SL (Figure 4A, grey dashed lines). In an attempt to strengthen this interaction, additional substitutions were made in RNA1 mutant 180 (Figure 3A), at the fifth and seventh positions of the TABS and/or at corresponding partner positions upstream of TP-SL, to generate mutants 181, 182 and 183, in which further pairing of the TABS was achieved via extending the TP-SL (Figure 4B). Due to the presence of this region within the 3′ end of the p88 ORF, amino acid changes in the RNA1 mutants were unavoidable; nonetheless, all mutants generated maintained replication competence. Testing of the three initial RNA1 mutants (181, 182 and 183) in protoplast infections in the absence of RNA2 revealed that RNA1 mutant 182 exhibited the highest levels of sg mRNA, at 33% the relative level observed in wt RNA1 and RNA2 coinfections (Figure 4C). In an effort to improve the sg mRNA yields from RNA1 mutant 182, the GC base pair in a potentially competing stem (Figure 4A, highlighted in brown) was converted to CC or AC mismatches, creating RNA1 mutants 280 and 281, respectively (Figure 4B, circled red nucleotides). These modifications, which were predicted to reduce the potential for opposing pairing, generated similar sg mRNA yields as mutant 182 (Figure 4C). Importantly, corresponding minus-strand sg mRNA intermediates were observed for mutants 182, 280 and 281, supporting their generation via a premature termination mechanism (Figure 4D). These concluding findings show that (i) it is possible to create a functional *cis*-acting attenuation structure via the introduction of a few substitutions into the TABS’s structural context and (ii) the *trans*-TA–TABS interaction does not contain any unique structural features that are essential for activating sg mRNA transcription.

Lastly, these results demonstrate that, as alluded to previously [21], it is plausible that RNA1 initially expressed its sg mRNA using a TA-independent, *cis*-acting, attenuation RNA structure and later, while in association with RNA2, evolved to a *trans*-acting, TA-dependent mechanism; perhaps involving an intermediate stage where both modes of transcriptional activation were partially active (Figure 4E). The ultimate transition to a *trans*-system provided clear benefits not afforded by the *cis*-system: (i) the coordination of CP production with RNA1 and RNA2 abundance [13] and (ii) a mechanism to co-package the two genome segments [19,31]. Thus, the proposed conversion in transcriptional regulatory mechanism would have yielded a more fit segmented virus and would classify the current TP-SL/TABS structure in RNA1 as a partial fossil of a past *cis*-acting attenuation RNA structure.

## References

[1] Sit T.L., Lommel S.A. (2010). Tombusviridae. Encyclopedia of Life Sciences.

[2] Blanco-Pérez M., Hernández C. (2016). Evidence supporting a premature termination mechanism for subgenomic RNA transcription in Pelargonium line pattern virus: Identification of a critical long-range RNA-RNA interaction and functional variants through mutagenesis. J. Gen. Virol..

[3] Wu B., Oliveri S., Mandic J., White K.A. (2010). Evidence for a premature termination mechanism of subgenomic mRNA transcription in a carmovirus. J. Virol..

[4] Lin H.X., Xu W., White K.A. (2007). A multicomponent RNA-based control system regulates subgenomic mRNA transcription in a tombusvirus. J. Virol..

[5] Wang S., Mortazavi L., White K.A. (2008). Higher-order RNA structural requirements and small-molecule induction of tombusvirus subgenomic mRNA transcription. J. Virol..

[6] Jiwan S.D., Wu B., White K.A. (2011). Subgenomic mRNA transcription in tobacco necrosis virus. Virology.

[7] Xu W., White K.A. (2008). Subgenomic mRNA transcription in an aureusvirus: Down-regulation of transcription and evolution of regulatory RNA elements. Virology.

[8] Xu W., White K.A. (2009). RNA-based regulation of transcription and translation of aureusvirus subgenomic mRNA1. J. Virol..

[9] Sztuba-Solińska J., Stollar V., Bujarski J.J. (2011). Subgenomic messenger RNAs: Mastering regulation of (+)-strand RNA virus life cycle. Virology.

[10] Jiwan S.D., White K.A. (2011). Subgenomic mRNA transcription in Tombusviridae. RNA Biol..

[11] Lin H.X., White K.A. (2004). A complex network of RNA-RNA interactions controls subgenomic mRNA transcription in a tombusvirus. EMBO J..

[12] Chkuaseli T., White K.A. (2020). Activation of viral transcription by stepwise largescale folding of an RNA virus genome. Nucleic Acids Res..

[13] Sit T.L., Vaewhongs A.A., Lommel S.A. (1998). RNA-mediated trans-activation of transcription from a viral RNA. Science.

[14] Xiong Z., Lommel S.A. (1989). The complete nucleotide sequence and genome organization of red clover necrotic mosaic virus RNA-1. Virology.

[15] Tajima Y., Iwakawa H.O., Kaido M., Mise K., Okuno T. (2011). A long-distance RNA-RNA interaction plays an important role in programmed -1 ribosomal frameshifting in the translation of p88 replicase protein of Red clover necrotic mosaic virus. Virology.

[16] Xiong Z., Kim K.H., Giesman-Cookmeyer D., Lommel S.A. (1993). The roles of the red clover necrotic mosaic virus capsid and cell-to-cell movement proteins in systemic infection. Virology.

[17] Basnayake V.R., Sit T.L., Lommel S.A. (2009). The Red clover necrotic mosaic virus origin of assembly is delimited to the RNA-2 trans-activator. Virology.

[18] Park S.H., Sit T.L., Kim K.H., Lommel S.A. (2013). The red clover necrotic mosaic virus capsid protein N-terminal amino acids possess specific RNA binding activity and are required for stable virion assembly. Virus Res..

[19] Basnayake V.R., Sit T.L., Lommel S.A. (2006). The genomic RNA packaging scheme of Red clover necrotic mosaic virus. Virology.

[20] Tatsuta M., Mizumoto H., Kaido M., Mise K., Okuno T. (2005). The red clover necrotic mosaic virus RNA2 trans-activator is also a cis-acting RNA2 replication element. J. Virol..

[21] Newburn L.R., White K.A. (2020). A trans-activator-like structure in RCNMV RNA1 evokes the origin of the trans-activator in RNA2. PLoS Pathog..

[22] White K.A., Morris T.J. (1994). Nonhomologous RNA recombination in tombusviruses: Generation and evolution of defective interfering RNAs by stepwise deletions. J. Virol..

[23] Cimino P.A., Nicholson B.L., Wu B., Xu W., White K.A. (2011). Multifaceted regulation of translational readthrough by RNA replication elements in a tombusvirus. PLoS Pathog..

[24] Vasa S.M., Guex N., Wilkinson K.A., Weeks K.M., Giddings M.C. (2008). ShapeFinder: A software system for high-throughput quantitative analysis of nucleic acid reactivity information. RNA.

[25] Reuter J.S., Mathews D.H. (2010). RNAstructure: Software for RNA secondary structure prediction and analysis. BMC Bioinform..

[26] Johnson P.Z., Kasprzak W.P., Shapiro B.A., Simon A.E. (2019). RNA2Drawer: Geometrically strict drawing of nucleic acid structures with graphical structure editing and highlighting of complementary subsequences. RNA Biol..

[27] Choi I.R., Ostrovsky M., Zhang G., White K.A. (2001). Regulatory activity of distal and core RNA elements in Tombusvirus subgenomic mRNA2 transcription. J. Biol. Chem..

[28] Fabian M.R., White K.A. (2006). Analysis of a 3′-translation enhancer in a tombusvirus: A dynamic model for RNA-RNA interactions of mRNA termini. RNA.

[29] Lafuente E., Ramos R., Martínez-Salas E. (2002). Long-range RNA-RNA interactions between distal regions of the hepatitis C virus internal ribosome entry site element. J. Gen. Virol..

[30] Guenther R.H., Sit T.L., Gracz H.S., Dolan M.A., Townsend H.L., Liu G., Newman W.H., Agris P.F., Lommel S.A. (2004). Structural characterization of an intermolecular RNA-RNA interaction involved in the transcription regulation element of a bipartite plant virus. Nucleic Acids Res..

[31] Newburn L.R., White K.A. (2019). Trans-Acting RNA-RNA Interactions in Segmented RNA Viruses. Viruses.

